# Development of an e-supported illness management and recovery programme for consumers with severe mental illness using intervention mapping, and design of an early cluster randomized controlled trial

**DOI:** 10.1186/s12913-016-1267-z

**Published:** 2016-01-19

**Authors:** Titus A. A. Beentjes, Betsie G. I. van Gaal, Peter J. J. Goossens, Lisette Schoonhoven

**Affiliations:** 1 Radboud university medical center, Radboud Institute for Health Sciences, IQ healthcare, Geert Groteplein 21, PO Box 9101, 6500 HB Nijmegen, The Netherlands; 2 Saxion University of Applied Science, School of Health, Deventer, The Netherlands; 3 Dimence Group Mental Health Care Centre, Deventer, The Netherlands; 4 University Centre for Nursing and Midwifery, Department of Public Health, Faculty of Medicine and Health Sciences, Ghent University, Ghent, Belgium; 5 GGZ-VS, Institute for Education of Clinical Nurse Specialist in Mental Health, Utrecht, The Netherlands; 6 Faculty of Health Sciences, University of Southampton, Southampton, UK

**Keywords:** Severe mental illness, E-Mental health, Illness management and recovery, Intervention mapping, Early randomized controlled trial

## Abstract

**Background:**

E-mental health is a promising medium to keep mental health affordable and accessible. For consumers with severe mental illness the evidence of the effectiveness of e-health is limited. A number of difficulties and barriers have to be addressed concerning e-health for consumers with severe mental illness. One possible solution might be to blend e-health with face-to-face delivery of a recovery-oriented treatment, like the Illness Management & Recovery (IMR) programme. This paper describes the development of an e-health application for the IMR programme and the design of an early clustered randomized controlled trial.

**Method/Design:**

We developed the e-IMR intervention according to the six-step protocol of Intervention Mapping. Consumers joined the development group to address important and relevant issues for the target group. Decisions during the six-step development process were based on qualitative evaluations of the Illness Management & Recovery programme, structured interviews, discussion in the development group, and literature reviews on qualitative papers concerning consumers with severe mental illness, theoretical models, behavioural change techniques, and telemedicine for consumers with severe mental illness. The aim of the e-IMR intervention is to help consumers with severe mental illness to involve others, manage achieving goals, and prevent relapse. The e-IMR intervention consists of face-to-face delivery of the Illness Management & Recovery programme and an e-health application containing peer-testimonials on videos, follow up on goals and coping strategies, monitoring symptoms, solving problems, and communication opportunities.

We designed an early cluster randomized controlled trial that will evaluate the e-IMR intervention. In the control condition the Illness Management & Recovery programme is provided. The main effect-study parameters are: illness management, recovery, psychiatric symptoms severity, self-management, quality of life, and general health. The process of the IMR program will be evaluated on fidelity and feasibility in semi-structured interviews with participants and trainers.

**Discussion:**

Intervention Mapping provided a systematic procedure for the development of this e-health intervention for consumers with severe mental illness and the preparation of an early randomized controlled trial.

**Trial Registration:**

The trial is registered in the Dutch Trial Register: NTR4772 .

## Background

Dutch policy makers proclaimed the development of e-mental health to be a cornerstone of their policy to keep mental health affordable and accessible in the future. E-mental health applications hold promise to expand access to care [1], and they are expected to be efficient both economically and socially [2]. Current applications are most frequently aimed at adults with depression or anxiety disorders and some interventions have demonstrated effectiveness in early trials [1].

For consumers with severe mental illness (SMI) the evidence of the effectiveness of e-health is limited [3]. According to Delespaul [4] consumers with SMI are diagnosed with a psychiatric disorder that causes—and is due to—serious impairments in social and/or occupational functioning, which last longer than at least a couple of years, and necessitates coordinated multidisciplinary care. E-health interventions for consumers with SMI are considered to have potential for delivering effective education [5, 6]. But, a number of difficulties and barriers have been addressed concerning e-health for consumers with SMI, e.g. cognitive impairments and lower technology experience [7]. Ben-Zeev et al. [3] advises that future development of e-health interventions must be coupled with examining barriers and possible solutions. One possible solution might be to blend face-to-face and e-health delivery of recovery-oriented treatment programmes. This blending is considered to be the most optimal practice to non-SMI consumers [8].

An evidence-based recovery-oriented intervention is the Illness Management & Recovery (IMR) programme [9], which has proven effectiveness in three random controlled trials in different countries [10–12]. The IMR programme is a standardized curriculum-based approach designed to provide consumers with SMI information and skills necessary for managing their illnesses effectively and working towards achieving personal recovery goals [9]. The educational material is a hard copy textbook organized in eleven modules (See Table 1). IMR was introduced in the Netherlands in 2009 and is mostly co-facilitated by a peer-support specialist and a psychiatric nurse.

We developed an e-health supported Illness Management & Recovery programme (e-IMR intervention), which blends the possibilities of e-health and the standard face-to-face delivery of the IMR programme. In this paper we describe the development of the e-IMR intervention and how we will evaluate this intervention.

**Table 1:** Eleven IMR Modules

1. Recovery Strategies	
2. Practical Facts about Mental Illnesses	
3. The Stress-Vulnerability Model	
4. Building Social Support	
5. Using Medication Effectively	
6. Drugs and Alcohol Use	
7. Reducing Relapses	
8. Coping with Stress	
9. Coping with Persistent Symptoms	
10. Getting Your Needs Met in the Mental Health System	
11. Healthy Lifestyles	

## Methods/Design

### Developing the e-IMR intervention

The development of the e-IMR intervention followed the six steps of the Intervention Mapping protocol, a systematic approach to develop health promotion interventions [13]. Intervention Mapping helps the development of more effective behavioural change interventions [14] and has successfully been used for the development of various health programmes [15–17]. Table 2 shows the six Intervention Mapping steps with their objectives and the used methods. During the development process we presented the results to five IMR-experts (see acknowledgement) and they confirmed the results.

**Table 2:** Intervention Mapping steps, objectives and methods

Steps		Objectives	Methods
1.	Needs Assessment	• Gain insight into health problems and underlying determinants of consumers with SMI	• Problems analyses using PRECEDE model Resources: o Qualitative literature o Internet forum discussions o IMR resources and literature o Development group discussions
2.	Preparing Matrices of Change Objectives	• Set intervention outcomes • Specify performance objectives and changeable determinants • Identify proximal change objectives	• Content analyses • Resources: o Qualitative literature o Internet forum discussion o IMR resources and literature o Development group discussions
3.	Selecting Theory-Informed Intervention Methods and Practical Implications	• Identify and select theoretical models • Select methods that address change objectives • Select evidence-based interventions and design of practical implications	• Content analyses • Resources: o Literature on behavioural change theories o IMR resources and literature o Qualitative IMR evaluation o Literature on telemedicine and SMI o BCT Classification
4.	Producing Program Components and Materials	• Compile an intervention	• Choosing an e-health platform partner • Constructing and pretesting the e-IMR intervention • Resources: o Literature on telemedicine and SMI
5.	Planning Program Adoption and Implementation	• Preparing implementation	• Resources: o Literature on telemedicine and SMI o Interviewing consumers on computer literacy (n = 52)
6.	Planning for Evaluation	• Setting up an evaluation plan	• Designing an early RCT • Resources: o The MRC guideline o Literature on questionnaires

*BCT* Behavioural Change Techniques, *IMR* Illness Management & Recovery programme, *MRC* Medical Research Council, *PRECEDE* Predisposing, Reinforcing and Enabling Constructs in Educational Diagnosis and Evaluation, *RCT* Random Controlled Trial, *SMI* Severe Mental Illness

### Step 1: Needs assessment

The intervention mapping protocol starts with assembling a development group to assure that the project addresses important and relevant issues for the target group [13]. Our group consisted of six consumers with SMI who previously completed the IMR programme, an informal caregiver, and two professionals acquainted with the IMR programme, of which one is a peer support specialist. Two researchers (TB, BVG) completed the group. The group members brought in their own knowledge, based on experience, profession, or science and thus contributed to group discussions and brainstorm sessions.

Further, the aim of this step is to get insight into health problems and underlying determinants of consumers with SMI. Therefore, we used a number of methods. (1) We reviewed literature on health problems of consumers with SMI. The MEDLINE, PsychINFO, EMBASE, and CINHAL databases were searched for articles between 2003–2013, retrieving 42 eligible papers. Only qualitative papers were included to be able to stay close to what consumers reported themselves. (2) We searched on Google for consumers’ testimonials in Dutch internet forums where people with SMI share their problems and needs with peers. (3) We studied IMR resources [9, 18]. (4) We discussed the findings in the development group.

**Fig. 1:**
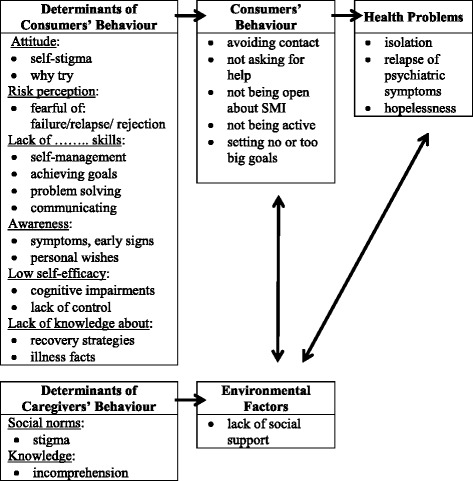
PRECEDE model of health problems of consumers with SMI

To analyse all the data, we used the PRECEDE model [19] to relate health problems to consumers’ health behaviour and their determinants, and environmental factors and their determinants. identified three important health problems that are influenced by the consumers’ behaviour and environmental factors, including caregivers’ behaviour and their determinants (See Fig. 1). The health problems of consumers with SMI are isolation, relapse of psychiatric symptoms, and hopelessness. Members of the development group stated that these problems are intertwined.

Isolation is described as feelings of exclusion and loneliness. Consumers report feelings of having no one to talk to about their problems and feelings of being misunderstood by other people. Consumers are susceptible for relapse of being overwhelmed by psychiatric symptoms like anxiety, depression, mania, and psychosis. This is related to an unstable balance between their biological vulnerability and stress. Problems with hopelessness are described as having a life that is marked with suffering and limitations, never-ending uncertainties, feelings of uselessness, and meaningless.

The underlying consumers’ behaviour that causes—and is due to—these health problems are avoiding contact with others, not asking for help, and not being open about issues related to their SMI, resignation such as choosing not to be active, and setting no goals at all or unrealistic goals for the future. These behaviours have determinants in common. The first determinant is attitude, like self-stigma and ‘why try’ [20]. Self-stigma includes feelings of shame, being different, or being the only one with a SMI. The ‘why try’ attitude is characterized by the question ‘why should I try?’, which affects goal-related behaviour. The second determinant is risk perception, like fear of being rejected by others, expecting to fail, and fearing relapse. The third determinant is the lack of skills, such as skills to cope with symptoms, skills to set and achieve recovery goals, problem-solving skills, and communicating skills, e.g. how to tell people about their condition. The fourth determinant is awareness. Consumers have difficulties in recognizing early warning signs and lost awareness of things they would like to achieve. The fifth determinant is self-efficacy, influenced by cognitive impairments, such as little concentration and attention, and the lack of control or mastery over one’s behaviour. The last determinant is lack of knowledge about the illness and recovery possibilities.

The main environmental factor that influences behaviour problems is lack of social support, such as lack of acknowledgement and ineffective encouragement. Often consumers get infeasible suggestions of how to solve problems. This is determined by the social norms related to stigma and lack of knowledge that causes incomprehension. Other people cannot understand the difficulties that come along with experiencing psychiatric symptoms.

### Step 2: Preparing matrices of change objectives

The second step aimed to identify the intervention objectives. First, we described the behavioural outcomes. Second, we formulated performance objectives and their changeable determinants of what consumers need to do to attain the outcomes. Next, we created a matrix by crossing performance objectives with determinants and change objectives. The change objectives describe what consumers need to learn to execute the performance objectives. For this purpose we studied the same resources as in the first step, and searched for descriptions of healthy behaviour that could be linked to the results of the first step and discussed in the development group.

As a result, we formulated three main behavioural outcomes of the intervention. The first behavioural outcome is having connections with other important people. The connected performance objective is learning to disclose their SMI. The second outcome is achieving personal recovery goals and relates to the objective planning and executing attainable steps towards recovery goals. The third outcome is reducing relapse of psychiatric symptoms, which relates to the objective coping actively with symptoms and stressors. Another performance objective is ‘arranging social support’, which relates to the second and third outcomes. Consumers learn to instruct others on what really supports them and what is not feasible for them. The determinants of the performance objectives are the same as in step 1. In order to influence the determinants we formulated proximal change objectives. Table 3 shows the matrix of the outcomes, performance objectives, determinants, and proximal change objectives.

**Table 3:** Matrix of behavioural outcomes, performance objectives, determinants, and proximal change objectives

Behavioural Outcomes	Performance Objectives	Personal Determinants and the Related Proximal Change Objectives				
		Knowledge	Perceived Norms	Attitude	Skills/Self-Efficacy	Awareness/Outcome Expectation
Having connections with other important people	Disclose about having a SMI	Lists people that respect him or her	Recognize others need information to understand his or her SMI	Acknowledge equality to others	Express confidence in ability to explain to others about his or her SMI	Expect disclosure will neutralize isolation
				Acknowledge stigma in others		
					Express confidence in ability to solve problems related to social relations	
Achieving personal recovery goals	Plans and executes attainable steps to achieve personal recovery goals	Defines what recovery means to him or her	Recognize people with SMI can achieve goals and have strength	Acknowledge it is worth trying to achieve goals	Express confidence in ability to break down long-term recovery goals into short-term goals and attainable steps	Estimate personal wishes and goals
		Identifies personal long-term recovery goals		Acknowledge personal pros and cons of being active		Monitor goal achievements
				Acknowledge there is hope for the future	Express confidence in ability to solve problems related to executing the steps	Expect that achieving goals will help coping with the illness
		Identifies short-term goals and attainable steps		Acknowledge that failure is common; not your fault		
Reducing relapse of psychiatric symptoms	Active coping (early) symptoms and stressors	Define correlation between stress and biological vulnerability, symptoms, and relapse	Recognize peers have similar symptoms and problems	Acknowledge that he or she is the expert on his or her SMI	Express confidence in ability to manage stressors actively according to relapse prevention plan	Monitor symptoms
						Expect active coping will prevent relapse and help to achieve goals
				Acknowledge his or her stress vulnerability	Express confidence in ability to solve problems related to coping with symptoms and stressors	Monitor successful coping strategies
		Lists personal (early) symptoms and stressors	Recognize peers are respectful people	Acknowledge personal pros and cons of active coping		
		Lists personal management strategies in a relapse prevention plan				
Achieving goals and reducing relapse	Arrange support regarding coping with symptoms and achieving goals	Lists the people that are able to support him or her		Acknowledge dependence (on others) that empowers	Express confidence in ability to explain (and ask for) what people can do to support them	Expect social support helps to achieve goals and cope actively

### Step 3: Selecting theory-informed intervention methods and practical applications

The third step aimed to identify and select theoretical models and evidence-based methods that could address the change objectives that were described in the second step. For this purpose we studied the literature on theoretical models of behavioural change [13, 21] and the underlying theories and methods of the IMR programme [9]. In the development group we discussed the findings of two qualitative evaluations of how consumers experienced the IMR programme [22, van Langen W, Beentjes TAA, van Gaal BGI, Nijhuis-van der Sanden R, Goossens PJJ: How the Illness Management and Recovery Programme Enhanced Recovery of Persons with Severe Mental Illness: A Qualitative Study, submitted]. Next we matched the change objectives with evidence-based methods using the manual for Behavioural Change Techniques Classification [23]. To select practical applications we studied peer-reviewed literature on telemedicine and SMI about examples of e-health interventions for consumers with SMI.

As a result, we concluded that the IMR programme is based on the theories Stress-Vulnerability Model and the Trans Theoretical Model (TTM) [9]. These theories are also applicable for e-IMR intervention. The Stress-Vulnerability Model [24] describes how the balance between stress and biological vulnerability can be disturbed and that consumers actively keep or restore the balance. This relates to the problem of susceptibility for relapse as described in step 1 and the active coping objectives as described in step 2. The TTM describes that the process of change includes increasing knowledge, raising awareness, changing attitudes, changing perceived norms, and increasing self-efficacy [25]. This process matches the content of the determinants of the problem as described in the first step and the determinants of the performance objectives as mentioned in Table 3.

As a result of discussing the components and applications of the e-IMR intervention, we will provide peer information and social comparison to affect consumers’ attitudes and perceived norms by applying peer testimonials on videos. Modelling will be used applying these peer testimonials that will show examples of how skills can be performed. Opening the intervention for important others will help consumers to disclose and involve others in order to get the proper social support by executing coping skills. Therefore, the intervention contains communication options with peers, trusted persons, and IMR trainers.

To enhance the consumers’ achievements of recovery goals we will use the methods of setting graded task of attainable steps and reinforcements of progress. In the intervention consumers will keep track of the goals and the goal achievements. To enhance self-efficacy consumers need to keep track of successful attempts of coping with symptoms. This enlarges the chance that the new behaviour will be used again. Setting graded tasks and weighing pros and cons will also be used in the problem-solving method. To raise awareness of symptoms and enhance insight into the course of their symptoms the intervention applies monitoring symptoms.

To get familiar with the applications, such as keeping track of goals and the problem-solving method and to maintain its use, the intervention will continuously provide these applications. We assume that repeated use will increase consumers’ confidence and ability to execute the performance objectives.

### Step 4: Producing program components and materials

The fourth step aimed to design the e-IMR intervention with components and materials that match the proximal change objectives and methods that were identified in the previous steps. For this purpose (1) we studied peer-reviewed literature on telemedicine and SMI to determine design issues, (2) we gathered information about information and communication technology (ICT) partners who could deliver the desired components on a protected e-health platform. Designing the e-IMR intervention was completed after pretesting the intervention thoroughly on legibility, usability, and bugs.

As a result, we chose an ICT partner because of their broad experience in developing e-mental health modules and their ability to provide login data. The website of the e-IMR intervention was designed according to the Flat Explicit Design Model in order to increase user-friendliness for people with cognitive impairments and lower technology experience [7]. Therefore, we avoided the use of mandatory fields, the need to scroll back and forth on the website, the need to go to different pages for additional content, and the use of large text fragments.

The e-IMR intervention uses the same routeing as the IMR programme: 11 modules (see Table 1), all together 56 chapters, for approximately 40 weekly sessions.

The e-IMR intervention uses a hard copy textbook along with the website in which the videos and practice at home assignment options are provided. At the end of the first module the goal follow-up system is introduced and provided in all later chapters. At the end of the second module and in all the later chapters, the coping skill follow-up system is provided. When the second module is completed, the consumers will be alerted via weekly e-mail to register their symptoms. The problem-solving method is presented as a practice at home assignment option in the last chapter of each module. Each chapter will finish with an evaluation of the chapter.

Within the intervention, the IMR trainer is able to start a discussion with the consumer. During face-to-face sessions the trainer will open the website and discuss the content of each session. Furthermore, the consumers select a trusted person with whom they will share the content of the e-IMR intervention. When the e-IMR intervention is provided via group sessions, the trainer will use a group e-mail to start a discussion with group members.

### Step 5: Planning program adoption and implementation

The fifth Intervention Mapping step aimed to prepare the adoption and implementation of the e-IMR intervention in the Netherlands. For this purpose we studied the literature on telemedicine for consumers with SMI to identify implementation barriers, and we conducted structured interviews with 52 Dutch consumers with SMI regarding the use of computers and the Internet.

As a result, we decided to implement the e-IMR intervention in institutions that already provide the IMR programme. These institutes are assembled in the Dutch IMR network. Saxion, University of Applied Sciences supports this network and educates trainers to carry out the IMR programme. This network facilitates the implementation and provides trainers that will be able to execute the e-IMR intervention after being instructed how to use the applications of the e-IMR intervention.

Implementation could not only be hindered by cognitive impairments and lower technology experience of consumers with SMI [7], but also by not having access to a computer. The results of the structured interviews showed that approximately 40 % of consumers with SMI had no computer, mostly because of financial problems. Therefore, we will assess the consumers’ computer skills before they enter the e-IMR intervention. When necessary we will help the consumer to search for a place where he or she can use a computer and support him or her to learn to use the website of the e-IMR intervention.

### Step 6: Planning for evaluation

The sixth and last step aimed to develop an evaluation plan to determine whether the e-IMR intervention contributes to the recovery process of consumers with SMI and matches the consumers’ preferences. For this purpose we studied the Medical Research Council guidance [26].

As a result, we decided to test the e-IMR intervention in an exploratory cluster randomized controlled trial with a twelve-month follow-up from baseline. This trial will not be powered as it will aim to (1) explore the potential effectiveness and effect-size, (2) to identify outcome measures that most likely capture consumers’ potential benefits of the e-IMR intervention, (3) to explore the actual use and added value, and (4) to evaluate continued participation or dropping out of the e-IMR intervention.

Participants in the experimental condition will receive the e-IMR interventions containing the use of the hard copy textbook and the programme components described in step 4. Participants in the control condition will receive the IMR programme only using the hard copy textbook. Both conditions will be provided in individual or group sessions. All participants will also receive care consisting of extensive in- and/or outpatient psychiatric services including case management and guideline-based psychiatric treatments.

Before randomization, we stratified institutions within types (outpatients/inpatients clinics). Due to risk of contamination, units that could contaminate each other were combined in one cluster. Because of high attrition rates in the IMR programme [27] and e-health [28] we need 50 participants in both conditions.

The inclusion period has been closed in October 2015. The follow-up will last until the end of October 2016 followed by the results at the end of December 2016.

#### Settings and eligibility

Settings are eligible if trained and experienced trainers provide the IMR programme. Consumers who are referred to the IMR programme by their clinician will be eligible to participate. Consumers that meet the following criteria will be included: above 18 years of age, capable of giving informed consent, and meeting the SMI criteria: having a diagnoses of schizophrenia, schizo-affective disorder, bipolar disorder, or major depression, with a duration of one year since onset, and a disability that is sufficiently severe to cause serious impairment of functioning in family responsibilities, occupation, and accommodation [29].

#### Data collection

At baseline we will collect the participant characteristics: age, gender, living situation, social economic status, diagnosis, time since diagnosis, previous treatment, latest relapse, relapse frequencies in the past, and computer/internet availability and literacy. Also at baseline we collect the trainer characteristics: profession, highest education, gender, age, years of experience in mental health, and the number of executed IMR programmes in the past.

At baseline and at twelve months the trainers will be interviewed and rated according to the IMR Fidelity Scale [30] to assure that the IMR programme is executed as it is meant to be.

To measure the effect we will examine the following parameters at baseline, six, and twelve months: illness management, recovery, psychiatric symptoms severity, self-management, quality of life, and general health. Respectively, we use the following measurements: (1) the clinician and consumer version of the Illness Management and Recovery Scales [31, 32], (2) the Mental Health Recovery Measure [33, 34], (3) the Brief Symptom Inventory [35, 36], (4) the Patient Activation Measure [37, 38], (5) the Manchester Short Assessment of Quality of Life [39], and (6) the RAND 36-Item Health Survey [40].

At 12 months we will gather login data on the frequency of participants’ use of the website of the e-IMR intervention. When participants and trainers completed the 12-month questionnaires, we will interview them qualitatively about the added value of the e-IMR intervention, the actual use and continued participation, or dropping out.

#### Analyses

Quantitative analyses will be executed according to the intention to treat principle. Multilevel regression with a random setting effect and repeated measures will be used to explore the effectiveness of the e-IMR intervention. Baseline measures and participants’ characteristics will be added as covariates. The relevance of the frequency of participants’ use of e-IMR intervention, consumer characteristics, trainer characteristics, and IMR Fidelity Scale ratings will be explored by performing sub-group analyses.

The process of interviewing and analysing the qualitative data will be guided by the modified method of Stevick–Colaizzi–Keen [41].

#### Ethics

The ethical approval for conducting the e-IMR trial was provided by the Committee on Research Involving Human Subjects, Arnhem-Nijmegen (NL49693.091.14). The trial is registered in the Dutch Trial Register (NTR4772).

## Discussion

In this paper we described the systematic development of the e-IMR intervention. This intervention blends the use of e-health and face-to-face delivery of the IMR programme in order to mitigate problems with isolation, relapse, and hopelessness of consumers with SMI. The e-IMR intervention will help these consumers to involve other important people, manage achieving personal recovery goals, and reduce relapse.

The focus on learning self-management skills and taking the lead in their treatment addresses development in the mental health policy in the Netherlands. E-mental health is a promising medium [2] and the e-IMR intervention matches the changes in the future profile of the nursing profession in the Netherlands [42]. Nurses need to focus on estimating what is necessary to support and teach the consumer self-management skills.

The use of the Intervention Mapping Protocol assured a systematic approach of the development of the intervention, including participation of the target population. In the sixth step of this protocol we designed an early-randomized controlled trial to evaluate the intervention. The use of an exploratory trial is advised in the Medical Research Counsel’s framework for the development and evaluation of complex interventions [26]. The choice of using the IMR programme in the control group allows evaluating the added value of the e-health components to this IMR programme. As Ben-Zeev et al. [3] advises this study is coupled with examining barriers and possible solutions. Therefore, this study will add new information to the body of knowledge in the field of e-health to consumers with SMI.

We conclude that the Intervention Mapping protocol provided a systematic procedure for the development of the e-IMR intervention for consumers with SMI. Estimating the added value of e-health will be possible because it is controlled by the standard IMR programme.

## Abbreviations

SMI: Severe mental illness
IMR: Illness management & recovery
e-IMR: Electronic illness management & recovery
PRECEDE: Predisposing, reinforcing and enabling constructs in educational diagnosis and evaluation
ICT: Information and communication technology
TTM: Trans theoretical model
